# Hub Genes Identification in a Murine Model of Allergic Rhinitis Based on Bioinformatics Analysis

**DOI:** 10.3389/fgene.2020.00970

**Published:** 2020-08-25

**Authors:** Le Chen, Le Shi, Yue Ma, Chunquan Zheng

**Affiliations:** ^1^Department of Otolaryngology-Head and Neck Surgery, Eye, Ear, Nose, and Throat Hospital, Fudan University, Shanghai, China; ^2^Shanghai Key Clinical Disciplines of Otorhinolaryngology, Shanghai, China

**Keywords:** bioinformatics analysis, allergic rhinitis, hub genes, WGCNA, Anxa1

## Abstract

This study aimed to identify allergic rhinitis (AR)-related hub genes and functionally enriched pathways in a murine model. Dataset GSE52804 (including three normal controls and three AR mice) was downloaded from Gene Expression Omnibus (GEO). Differentially expressed genes (DEGs) were identified. Gene ontology (GO), Kyoto Encyclopedia of Genes and Genomes (KEGG) pathways, and protein–protein interaction (PPI) analyses of DEGs were performed to identify the hub genes in AR. The DEGs were classified into different modules by using the weighted gene co-expression network analysis (WGCNA). Moreover, to verify the potential hub genes, nasal mucosa tissues were obtained from murine AR models (*n* = 5) and controls (*n* = 5), and qRT-PCR and Western blot were performed. In this study, a total of 634 DEGs were identified. They were significantly enriched in 14 GO terms, such as integral component of membrane, plasma membrane, and G-protein-coupled receptor signaling pathway. Meanwhile, there were eight terms of KEGG pathways significantly enriched, such as Olfactory transduction, Cytokine–cytokine receptor interaction, and TNF signaling pathway. The top 10 hub genes (Rtp1, Rps27a, Penk, Cxcl2, Gng8, Gng3, Cxcl1, Cxcr2, Ccl9, and Anxa1) were identified by the PPI network. DEGs were classified into seven modules by WGCNA. According to qRT-PCR validation of the five genes of interest (Rtp1, Rps27a, Penk, Cxcl2, and Anxa1), the expression level of Rtp1 mRNA was significantly decreased in the AR group compared with the control group, while there are enhanced Rps27a, Penk, Cxcl2, and Anxa1 mRNA expressions in the AR mice group compared with the control group. Western blot was also performed to further explore the expression of Anxa1 in the protein level, and the results showed a similar expression trend.

## Introduction

Allergic rhinitis (AR) is a common chronic hyperresponsive upper respiratory disease that affects all age groups (Wallace and Dykewicz, 2017). It is a kind of IgE-mediated atopic disease (Greiner et al., 2011). The characteristic symptoms include nasal pruritus, sneezing, and nasal congestion, which lead to a decreased quality of life (Jaruvongvanich et al., 2016; Li et al., 2018). In addition, AR is associated with numerous comorbidities such as allergic conjunctivitis and asthma (Blome et al., 2020). With the increasing incidence of AR in recent decades, further exploration is required to investigate the pathogenesis and biological characteristic of AR.

Gene Expression Omnibus (GEO) integrated the independent published datasets in recent years, which enlarged the sample size and benefited further research (Li and Xu, 2019). Weighted gene co-expression network analysis (WGCNA) is a novel systematic biology approach to determine the correlations among genes. This method has been widely applied to investigate highly correlated gene expression modules across microarray samples (Wang et al., 2019) and to find the hub genes involved in many complicated diseases (Li et al., 2016; Riquelme Medina and Lubovac-Pilav, 2016; Ma et al., 2017; Chen et al., 2018; Shao et al., 2018; Tang et al., 2018; Xu et al., 2018). In the present study, we performed WGCNA to investigate the biological mechanisms underlying AR. In addition, Gene Ontology (GO), Kyoto Encyclopedia of Genes and Genomes (KEGG), and protein–protein interaction (PPI) analyses were also applied.

To the best of our knowledge, this is the first study to integrate bioinformatics analyses and animal models to investigate the pathogenesis of AR.

## Materials and Methods

### Microarray Data Processing

Series matrix files of GSE52804 were downloaded from the GEO^1^ database to identify the hub genes related to AR. The platform of the GSE52804 dataset was GPL6246 (Affymetrix Mouse Gene 1.0 ST Array). This dataset included three AR mice models and three normal controls (BALB/c; male; 6–8 weeks) (Supplementary Table S1). Mice in the AR group were sensitized and challenged with ovalbumin (OVA), while normal saline was used in the control group. They were sacrificed 2 h after the last challenge.

### Differentially Expressed Gene Identification

The raw microarray data of GSE52804 were normalized by R software (version 3.5.2, United States)^2^. The “impute” package^3^ was carried out to fill in missing values. To identify the differentially expressed genes (DEGs) between the AR group and the control group, the “limma” package (Ritchie et al., 2015) in R software was applied. | log FC| (fold change) > 0.5 and adj. *p* < 0.05 were considered statistically significant.

### Functional Enrichment Analysis

To further explore the function of the DEGs in AR, we used the database for Annotation, Visualization, and Integrated Discovery (DAVID)^4^ to perform the GO and KEGG pathway analyses (Ogata et al., 1999; Ashburner et al., 2000). DAVID is an online bioinformatics database that is commonly used to generate systematic functional annotations about genes and proteins (Zeng et al., 2019). GO analysis consists of molecular function (MF), cellular component (CC), and biological processes (BP) analyses. The cutoff in GO analysis was set as false discovery rate (FDR) < 0.05, and in KEGG analysis, it was *p* < 0.05.

### PPI Network Construction

We constructed the PPI network of DEGs by the Search Tool for the Retrieval of Interacting Genes (STRING)^5^. The cutoff point of correlation between proteins was set as greater than 0.9, and Cytoscape software (version 3.7.1, UAS) (Shannon et al., 2003) was used to visualize the results. Moreover, the top 10 hub genes of connectivity were identified by cytohubba, a plug-in of Cytoscape software (Chin et al., 2014).

### WGCNA of DEGs Between AR and Control Group

Weighted gene co-expression network analysis was used to identify the potential function and clusters highly correlated genes of DEGs in AR. The DEG co-expression modules were constructed by the “WGCNA” package in R software (Langfelder and Horvath, 2008). The topological matrix was constructed by TOM (topological overlap measure) (Yip and Horvath, 2007). To group similar modules, the threshold for cut height was set as 0.6, and the minimum number of genes in modules was 20.

### Murine AR Model

To verify the potential target genes identified by bioinformatics analysis, murine models were applied. Six-week old male BALB/c mice were obtained from SLAC Laboratory Animal (Shanghai, China) and raised in a SPF (specific pathogen free) environment in the Department of Laboratory Animal Science of Shanghai Medical college of Fudan University. Mice experimental protocol was approved by the Animal Care and Use Committee of Fudan University. Mice were randomly divided into two groups (*n* = 5 per group): the AR group and normal control group. AR murine models were induced according to the previously established protocol (Shi et al., 2017; Ma et al., 2018a). Briefly, in the AR group, mice were sensitized by intraperitoneal injection of 0.2 ml suspension, which contains 20 mg/ml of aluminum hydroxide (Sigma, United States) and 500 μg/ml of OVA (Sigma, United States) on Day 1, Day 8, and Day 15. Then, they were challenged by intranasal instillation with 20 μl of OVA (40 mg OVA dissolved in 1 ml of normal saline) daily from Day 22 to Day 28, while in the normal control group, mice were sensitized and challenged with normal saline. After the final stimulation, the nasal symptoms were observed for 15 min, which include frequency of sneezing and nose rubbing behavior.

### ELISA

Mouse blood was obtained from post-glomus venous plexus after anesthetized and centrifuged for 15 min at 3000 rpm. To evaluate the state of inflammation, serum OVA-specific IgE was detected using specific ELISA kit (Weiao, China), and each sample was analyzed in triplicate.

### RNA Extraction and Quantitative Reverse Transcription-Polymerase Chain Reaction (qRT-PCR) Analyses

Nasal mucosa tissues were harvested under a microscope. Total RNA was extracted from nasal mucosa tissues by TRIzol reagent (Invitrogen, United States). The purity and integrity of RNA were measured using a NanoDrop ND-2000 (Thermo, United States). PrimeScript RT Master mix (TaKaRa, China) was applied to synthesize complementary DNA (cDNA), and SYBR Premix Ex Taq^TM^ (Takara, China) was used for qRT-PCR by ABI PRISM 7500 Sequence Detection System (Applied Biosystems, United States). Both target genes and GAPDH were amplified in separate wells in triplicate. The qRT-PCR primers were designed and synthesized by Sangon Biotech (Shanghai, China). BLAST (Basic Local Alignment Search Tool from the NCBI)^6^ was applied to verify the primer design and to ensure the specificity of the primers. Primer sequences used are shown in Table 1. The relative expression level of mRNA was calculated by the 2−ΔΔCT method (Ma et al., 2018b).

**TABLE 1:** Primers used in this study.

Primers	Sequence (5′ → 3′)
Rtp1 (F)	GCTGCCCTGCCTTACACTTAC
Rtp1 (R)	CACCTGTGGTCACACTCTTAC
Rps27a (F)	ACTCCCAAGAAGAACAAGCATA
Rps27a (R)	CAGTAAGTCAGACAACACTTGC
Penk (F)	GGACTGCGCTAAATGCAGCTA
Penk (R)	GAAGCCTCCGTACCGTTTCAT
Cxcl2 (F)	CCAACCACCAGGCTACAGG
Cxcl2 (R)	GCGTCACACTCAAGCTCTG
Anxa1 (F)	ATGTATCCTCGGATGTTGCTGC
Anxa1 (R)	TGAGCATTGGTCCTCTTGGTA
GAPDH (F)	AGGTCGGTGTGAACGGATTTG
GAPDH (R)	TGTAGACCATGTAGTTGAGGTCA

### Western Blot (WB) Analysis

**FIGURE 1:**
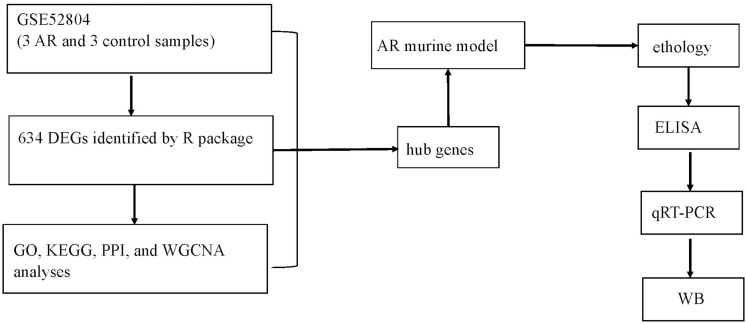
Workflow diagram of data collection and analysis.

Protein samples (30 μg) obtained from nasal mucosa tissues were separated by using 10% sodium dodecyl sulfate polyacrylamide gel electrophoresis (SDS-PAGE). Then, proteins were transferred to polyvinylidene difluoride (PVDF) membranes (Millipore, United States) according to the manufacturer’s guidelines for incubation with anti-ANXA1 antibody (1:1000, Abcam, United Kingdom). Meanwhile, the expression of β-actin (1:1000, Abcam, United Kingdom) was performed as an internal control. Image J software (NIH, United States) was applied to analyze data, which was shown as densitometry units. The workflow of this study is shown in Figure 1.

### Statistical Analysis

Differentially expressed genes identification and WGCNA were performed by R software (version 3.5.2, United States). Results were presented as the mean ± standard deviation (SD). GraphPad Prism software (version 7.0, United States) was applied to statistical analyses. *p*-value < 0.05 was considered statistically significant.

## Results

### DEGs Between the AR and Control Group

**FIGURE 2:**
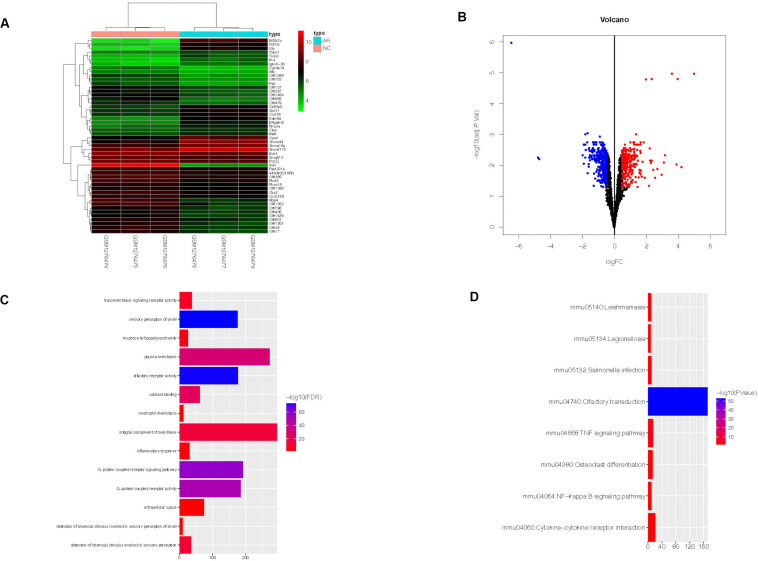
Heatmap, volcano plot, GO, and KEGG functional enrichment results of 634 DEGs in AR. The *x*-axis represents gene count and the *y*-axis represents GO or KEGG terms. **(A)** The top 50 DEGs identified by R software. The highly expressed genes of the AR group compared with the control group were represented by the red areas and the lowly expressed genes were represented by the green areas. **(B)** Red dots represent the up-regulated genes and blue dots represent the down-regulated genes of the AR group compared with the control group. **(C)** GO functional enrichment result of DEGs. **(D)** KEGG functional enrichment result of DEGs. DEGs, differentially expressed genes; AR, allergic rhinitis. GO, Gene Ontology; KEGG, Kyoto Encyclopedia of Genes and Genomes.

In dataset GSE52804, a total of 634 DEGs were identified between the AR group and the control group by R software (adj. *p* < 0.05, |logFC| > 0.5) (Supplementary Table S2), and the top 50 genes are shown in the heatmap (Figure 2A). There were 294 up-regulated genes and 340 down-regulated genes (Figure 2B).

### Functional Enrichment Results

DAVID online tool was used to perform GO and KEGG analyses and to identify the biological functions of the 634 DEGs in AR. GO analysis revealed that the DEGs were significantly enriched in 14 GO terms. They were mainly enriched in an integral component of membrane, plasma membrane, and G-protein-coupled receptor signaling pathway (Figure 2C). Meanwhile, KEGG analysis revealed that they were significantly enriched in eight KEGG terms, such as Olfactory transduction, Cytokine–cytokine receptor interaction, and TNF signaling pathway (Figure 2D).

### PPI Network Results

**FIGURE 3:**
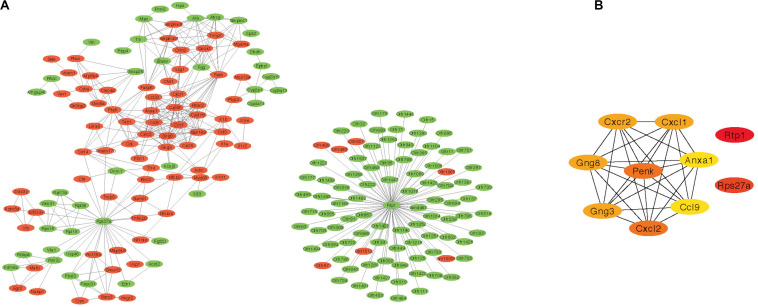
Protein–protein interaction network results and the top 10 hub genes. **(A)** PPI network of DEGs created by STRING (red represent highly expressed genes in AR and green represent lowly expressed genes). **(B)** The top 10 hub genes identified by cytohubba. PPI, protein–protein interaction; DEG, differentially expressed gene; AR, allergic rhinitis.

The PPI network of DEGs was constructed based on STRING database. The correlation between proteins was set as >0.9 score and then 602 nodes and 486 edges were produced (Figure 3A). Furthermore, the cytohubba plug-in of Cytoscape was applied, and the top 10 hub nodes (Rtp1, Rps27a, Penk, Cxcl2, Gng8, Gng3, Cxcl1, Cxcr2, Ccl9, and Anxa1) were obtained (Figure 3B).

### WGCNA Results

**FIGURE 4:**
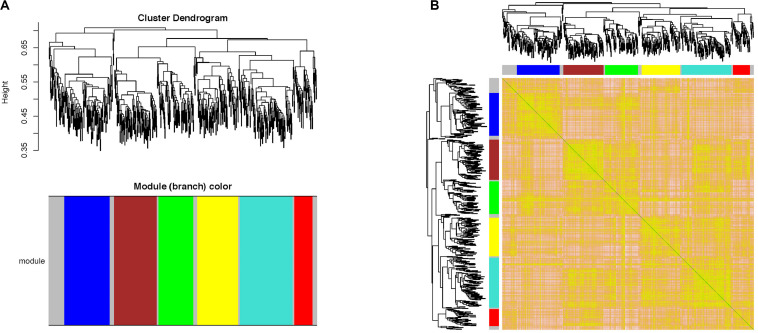
Weighted gene co-expression network analysis of the 634 DEGs in AR. **(A)** Clustering dendrogram of DEGs related to AR. **(B)** Network heatmap plot in the co-expression modules. WGCNA, weighted gene co-expression network analysis; DEGs, differentially expressed genes; AR, allergic rhinitis.

“WGCNA” package in R software was performed to merge the coherent expression genes into modules (Pu et al., 2020). Then, seven co-expression modules were obtained (Figure 4). To explore the biological function of genes corresponding to each module, GO functional enrichment analysis was applied and the most significantly enriched terms in each module are shown in Table 2.

**TABLE 2:** Gene ontology (GO) analysis results of modules constructed by WGCNA.

Module	GO_Term	*p*-value
Yellow	Extracellular exosome	9.25E-04
Turquoise	Olfactory receptor activity	4.99E-65
Red	G-protein-coupled receptor activity	3.04E-07
Brown	Cellular response to lipopolysaccharide	1.22E-05
Blue	Inflammatory response	2.39E-09
Green	Plasma membrane	3.74E-06
Gray	Defense response to bacterium	0.023411247

### Ethology and ELISA Results of the Mice Model

**FIGURE 5:**
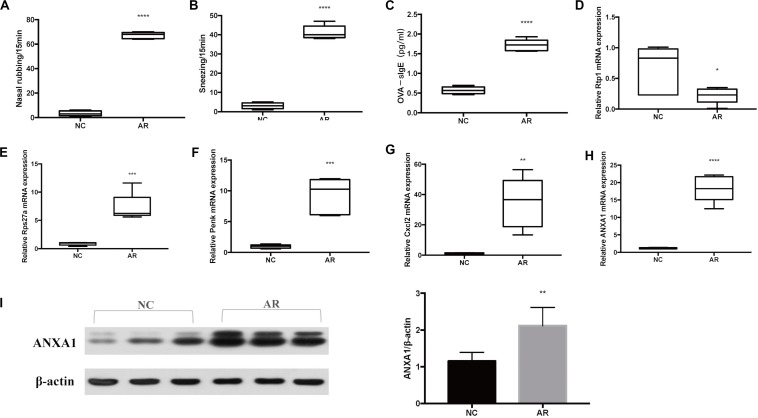
Murine model results. **(A)** Symptom score of sneezing. **(B)** Symptom score of nose rubbing. **(C)** Serum OVA-specific IgE was assayed by ELISA. The mRNA level of **(D)** Rtp1, **(E)** Rps27a, **(F)** Penk, **(G)** Cxcl2, and **(H)** Anxa1. **(I)** Western blot results of ANXA1 of nasal tissues and the densitometric analysis result. ****p < 0.0001, ***p < 0.001, **p < 0.01, *p < 0.05.

Sneezing was characterized by a sudden expiration, and nose rubbing was characterized by scratching of the nose with either one or both of the paw. Symptoms of sneezing and nose rubbing in the AR group and the normal control group were observed, and they were more severe in the AR mice model than in the control mice model (*p* < 0.0001) (Figures 5A,B). The ELISA result of serum OVA-specific IgE is shown in Figure 5C. Serum OVA-specific IgE increased significantly in the AR group (*p* < 0.0001).

### qRT-PCR Validation

To verify that the target genes are potentially involved in the pathogenesis of AR, we analyzed relative expression in nasal mucosa tissues of five AR mice and five control mice by qRT-PCR. According to the results of qRT-PCR of the five genes of interest (Rtp1, Rps27a, Penk, Cxcl2, and Anxa1), the expression level of Rtp1 mRNA was significantly decreased in the AR group compared with the control group (*p* < 0.05), while the Rps27a, Penk, Cxcl2, and Anxa1 mRNA expression levels were significantly increased in the AR group (Figures 5D–H).

### Western Blot Validation

Western blot was performed to further explore the expression of Anxa1 in the protein level. We can see that the expression of Anxa1 was significantly higher in the AR group than in the control group (Figure 5I).

## Discussion

Allergic rhinitis is a complex chronic inflammatory disease of the upper respiratory system (Ebrahim et al., 2019). It is a global health problem that impairs the quality of life and increases health care costs (Greiner et al., 2011; Cheng et al., 2018). However, despite a lot of effort have been made, the pathogenesis of AR is still not completely known and needs further study. In recent years, with the development of bioinformatics analysis and high-throughput microarray technology, microarray analyses have been widely used in many complicated diseases, but few related to AR. This system method provides a novel way to deeply understand the molecular mechanism of AR and allows us to identify the hub genes.

In this study, we downloaded dataset GSE52804, which includes three AR mice models and three control mice models. Then, a total of 634 DEGs were identified, including 294 up-regulated genes and 340 down-regulated genes. As we can see, the olfactory receptors account for a large part of the top 50 DEGs (Figure 2A). The olfactory receptor proteins are members of G-protein-coupled receptors (GPCR). They interact with odorant molecules in the nose, and the ectopic receptors show potential diagnostic and therapeutic applications in wounds, asthma, and cancers (Lee et al., 2019). A recent study has shown that AR resulted in significant olfactory receptor neuron loss and caused olfactory loss in the AR mice model (Liang et al., 2019).

According to GO analysis, DEGs were mainly enriched in an integral component of membrane, plasma membrane, and G-protein-coupled receptor signaling pathway. KEGG pathway analysis showed that the DEGs were mainly enriched in Olfactory transduction, Cytokine–cytokine receptor interaction, and TNF signaling pathway. Previous research suggested that TNF-α associated with increased intestinal epithelial permeability, and the HDM-sensitized mice model showed increased nasal permeability (Steelant et al., 2016; Ordovas-Montanes et al., 2018). Many studies suggest that GPER plays an important role in AR (Luginina et al., 2019; Wang et al., 2020). Meanwhile, the PPI network was applied to explore the interaction of proteins and identify the hub genes. The top 10 hub genes (Rtp1, Rps27a, Penk, Cxcl2, Gng8, Gng3, Cxcl1, Cxcr2, Ccl9, and Anxa1) were calculated by the cytohubba plug-in of Cytoscape. GO functional analysis was performed in the seven modules created by WGCNA (Table 2).

According to qRT-PCR results, the expression level of Rtp1 mRNA was significantly decreased in the AR group compared with the control group (*p* < 0.05). A previous study showed that Rtp1 (Receptor Transporting Protein 1) strongly expressed in the peripheral olfactory organs (Saito et al., 2004; Sharma et al., 2017), but as far as we know, there was no Rtp1-related study on AR, which is worth further investigation. By combining the above bioinformatics analysis results with the direction of our laboratory research, Anxa1 (Annexin A1) aroused our great interest in deeper exploration. To further understand the characteristics of Anxa1, related literature was extensively reviewed and comprehensively analyzed. Anxa1 is a 37-kDa calcium-binding protein, which is expressed in macrophages, monocytes, neutrophils, and epithelial cells (Leoni and Nusrat, 2016). It is a glucocorticoid-regulated protein and interacts with FPR2 (Corminboeuf and Leroy, 2015). FPR2 is a unique G-protein-coupled receptor, which is critical to the resolution of inflammation (Cooray et al., 2013). Previous studies have reported that Anxa1 and FPR2 play an important role in the pathogenesis of asthma (Perucci et al., 2017; Boeck et al., 2018; Lee et al., 2018). Anxa1 can reduce inflammation by regulating neutrophil transmigration, promoting neutrophil apoptosis and efferocytosis, inducing tissue repair, and recruiting monocytes (Maderna et al., 2005; Vago et al., 2012; Sugimoto et al., 2019; Machado et al., 2020). It displayed protective effects in many diseases, including allergic conjunctivitis, atopic dermatitis, and other diseases (Marmorato et al., 2019; Parisi et al., 2019; Senchenkova et al., 2019). However, the specific role of the Anxa1 in AR remained to be determined. To further evaluate the role of the Anxa1 gene in the pathogenesis of AR, experimental murine models were induced. The qRT-PCR results showed that the expression of Anxa1 mRNA in nasal mucosa was significantly up-regulated in the AR group (*p* < 0.0001) (Figure 5H). The expression trend of Anxa1 is similar to dataset GSE52804. Furthermore, the WB results showed that the expression of Anxa1 protein was significantly up-regulated in the AR group (*p* < 0.01) (Figure 5H). As far as we know, there is no study that prove that the Anxa1 gene may relate to the pathogenesis of AR. However, some relevant studies in asthma suggest that Anxa1 may play a role in AR (Cui and Yang, 2018; Lee et al., 2018; Ibrahim et al., 2020).

In general, this study was performed to identify the hub genes and pathways involved in AR by bioinformatics analysis. The hub gene was verified by murine models. It is reasonable to suppose that Anxa1 is important in the pathogenic progress of AR, and it deserves further research. Interestingly, genes associated with olfactory activity may also be relevant in AR, which indicates a new research direction of our laboratory.

## Data Availability Statement

All datasets generated for this study are included in the article/Supplementary Material.

## Ethics Statement

The animal study was reviewed and approved by Animal Care and Use Committee of Fudan University.

## Author Contributions

LC designed the study, participated in the experiments, analyzed the results, and drafted the manuscript. LS participated in model induction and analyzed the results. YM analyzed the results and participated in the experiments. CZ revised the manuscript. All authors read and approved the final manuscript.

## Conflict of Interest

The authors declare that the research was conducted in the absence of any commercial or financial relationships that could be construed as a potential conflict of interest.

## Footnotes

1 http://www.ncbi.nlm.nih.gov/geo

2 https://cran.r-project.org/

3 http://bioconductor.org/packages/release/bioc/html/impute.html

4 https://david.ncifcrf.gov/

5 http://string-db.org/cgi/input.pl

6 https://www.ncbi.nlm.nih.gov/tools/primer-blast/index.cgi?LINK_LOC=BlastHome
