# Supplementary material for: Hub Genes Identification in a Murine Model of Allergic Rhinitis Based on Bioinformatics Analysis
Source: Front Genet. 2020 Aug 25;11:970. doi: 10.3389/fgene.2020.00970 (PMC7477359; doi:10.3389/fgene.2020.00970)
Supplement: Supplementary file 1 [file Data_Sheet_1.DOCX]

1. DEGs identification

library(limma)

library("impute")

geo_data<-read.table("data.txt",sep="\t",header=T)

geo_data<-as.matrix(geo_data)

rownames(geo_data)=geo_data[,1]

geo_exp<-geo_data[,2:ncol(geo_data)]

dimnames<-list(rownames(geo_exp),colnames(geo_exp))

geo_exp<-matrix(as.numeric(as.matrix(geo_exp)),nrow=nrow(geo_exp),dimnames=dimnames)

mat=impute.knn(geo_exp)

geo_data=mat$data

geo_data=avereps(geo_data)

pdf("raw_box.pdf")

boxplot(geo_data,col = "green",xaxt = "n",outline = F)

dev.off()

geo_data=normalizeBetweenArrays(as.matrix(geo_data))

pdf("normal_box.pdf")

boxplot(geo_data,col = "red",xaxt = "n",outline = F)

dev.off()

class <- c(rep("normal",3),rep("treatment",3))

design <- model.matrix(~0+factor(class))

colnames(design) <- c("normal","treatment")

fit <- lmFit(geo_data,design)

cont.matrix<-makeContrasts(treatment-normal,levels=design)

fit1 <- contrasts.fit(fit, cont.matrix)

fit1 <- eBayes(fit1)

allgene<-topTable(fit1,adjust='fdr',number=100000)

write.table(allgene,"allgene.xls",sep="\t",quote=F)

normaldata<-allgene[order(allgene$logFC),]

normaldata1<-rbind(Gene=colnames(normaldata),normaldata)

write.table(normaldata,"normaldata.txt",sep="\t",quote=F,col.names=F)

diffgene <- allgene[with(allgene, (abs(logFC)>=0.5 & adj.P.Val < 0.05 )), ]

write.table(diffgene,"diffgene.xls",sep="\t",quote=F)

Upgene <- allgene[with(allgene, (logFC>=0.5 & adj.P.Val < 0.05 )), ]

write.table(Upgene,"upgene.xls",sep="\t",quote=F)

Downgene <- allgene[with(allgene, (logFC<=(-0.5) & adj.P.Val < 0.05 )), ]

write.table(Downgene,"down.xls",sep="\t",quote=F)

diffexp=geo_data[rownames(diffgene),]

diffexp1=rbind(id=colnames(diffexp),diffexp)

write.table(diffexp1,"diffexp.txt",sep="\t",quote=F,col.names=F)

inputfile_vol="vol.txt"

mydata<-read.table(inputfile_vol,header=T,row.names=1,check.names=F)

down <- mydata[mydata$logFC <= -0.5 & mydata$adj.P.Val<0.05,]

up <- mydata[mydata$logFC >= 0.5 & mydata$adj.P.Val<0.05,]

no <- mydata[(mydata$logFC > -0.5 & mydata$logFC <0.5),]

down<- transform(down,adj.P.Val=-log10(down$adj.P.Val))

up<- transform(up,adj.P.Val=-log10(up$adj.P.Val))

no<- transform(no,adj.P.Val=-log10(no$adj.P.Val))

pdf("vol1.pdf")

xm=max(abs(mydata$logFC))

ym=max(-log10(mydata$adj.P.Val))

plot(no$logFC,no$adj.P.Val,xlim = c(-xm,xm),ylim=c(0,ym),col="black",pch=16,cex=0.9,main = "Volcano",xlab = "logFC",ylab="-log10(adj.P.Val)")

points(up$logFC,up$adj.P.Val,col="red",pch=16,cex=0.9)

points(down$logFC,down$adj.P.Val,col="blue",pch=16,cex=0.9)

abline(v=0,lwd=2)

dev.off()

inputheatmap="diffexp.txt"

library(pheatmap)

data<-read.table(inputheatmap,sep="\t",header=T,row.names=1,check.names=F)

data<-data[1:50,]

pdf("heatmap.pdf")

annotation=read.table("group.txt",sep="\t",header=T,row.names=1)

pheatmap(data,display_numbers = F,annotation=annotation,fontsize_row=7,fontsize_col=10,cluster_cols = T,cluster_rows = T,color = colorRampPalette(c("green", "black", "red"))(50))

dev.off()

2. WGCNA of DEGs

library(WGCNA)

inputdata1="data.txt"

data0=read.table(inputdata1,sep="\t",row.names=1,header=T,check.names=F,quote="!")

datSummary=rownames(data0)

datExpr = t(data0)

no.samples = dim(datExpr)[[1]]

dim(datExpr)

powers1=c(seq(1,10,by=1),seq(12,20,by=2))

RpowerTable=pickSoftThreshold(datExpr, powerVector=powers1)[[2]]

cex1=1

par(mfrow=c(1,2))

pdf("beta.pdf")

plot(RpowerTable[,1], -sign(RpowerTable[,3])*RpowerTable[,2],xlab="

Soft Threshold (power)",ylab="Scale Free Topology Model Fit,signed R^2",type="n")

text(RpowerTable[,1], -sign(RpowerTable[,3])*RpowerTable[,2], labels=powers1,cex=cex1,col="red")

abline(h=0.85,col="red")

plot(RpowerTable[,1], RpowerTable[,5],xlab="Soft Threshold (power)",ylab="Mean Connectivity", type="n")

text(RpowerTable[,1], RpowerTable[,5], labels=powers1, cex=cex1,col="red")

dev.off()

beta1=8

Connectivity=softConnectivity(datExpr,power=beta1)

pdf("scalefree.pdf",15,10)

par(mfrow=c(1,1))

scaleFreePlot(Connectivity, main=paste("soft threshold, power=",beta1), truncated=T)

dev.off()

ConnectivityCut = 1000

ConnectivityRank = rank(-Connectivity)

restConnectivity = ConnectivityRank <= ConnectivityCut

ADJrest = adjacency(datExpr[,restConnectivity], power=beta1)

dissTOM=TOMdist(ADJrest)

hierTOM = hclust(as.dist(dissTOM),method="average")

colorh1= cutreeStaticColor(hierTOM,cutHeight = 0.6, minSize = 20)

pdf("module.pdf")

par(mfrow=c(2,1),mar=c(2,4,1,1))

plot(hierTOM, main="Cluster Dendrogram", labels=F, xlab="", sub="")

plotColorUnderTree(hierTOM,colors=data.frame(module=colorh1))

title("Module (branch) color")

dev.off()

pdf("TOM.pdf")

TOMplot(dissTOM , hierTOM, colorh1, terrainColors=TRUE)

dev.off()

pdf("cmd.pdf")

cmd1=cmdscale(as.dist(dissTOM),3)

pairs(cmd1, col=as.character(colorh1), main="MDS plot")

dev.off()

library(scatterplot3d)

pdf("3d.pdf")

par(mfrow=c(1,1), mar=c(4,3,2,3)+0.1)

scatterplot3d(cmd1,color=colorh1,angle=250,

xlab="Scaling Axis 1", ylab="Scaling Axis 2", zlab="Scaling Axis 3")

dev.off()

datME=moduleEigengenes(datExpr[,restConnectivity],colorh1)[[1]]

dissimME=1-(t(cor(datME, method="p")))/2

hclustdatME=hclust(dist(dissimME), method="average" )

pdf("modul_cluster.pdf")

par(mfrow=c(1,1))

plot(hclustdatME, main="Clustering tree based on the module eigengenes of modules")

dev.off()

pdf("modul_cor.pdf")

pairs(datME)

dev.off()

modul<-signif(cor(datME, use="p"), 2)

write.table(modul,"modul_cor.txt",sep="\t",quote=F)

datME=moduleEigengenes(datExpr,colorh1)[[1]]

color1=rep("grey",dim(datExpr)[[2]])

color1=as.character(colorh1)

datKME=signedKME(datExpr, datME)

datout=data.frame(datSummary, colorNEW=color1,datKME )

write.table(datout, "gene_module.xls", sep="\t", row.names=F,quote=F)
